# Comparative efficacy of intravenous immunoglobulin and protein A immunoadsorption in severe anti-NMDAR encephalitis

**DOI:** 10.3389/fimmu.2025.1602047

**Published:** 2025-08-12

**Authors:** Xuhui Tang, Yu Liu, Gaoya Zhou, Ewen Tu, Cheng Yu, Meishan Xiong, Cong Lin

**Affiliations:** ^1^ Department of Neurology, The Second People’s Hospital of Hunan Province, Brain Hospital of Hunan Province, Changsha, China; ^2^ Department of Neurology, College of Clinical Medicine, Hunan University of Chinese Medicine, Changsha, China; ^3^ Department of Neurology, The Fourth Hospital of Changsha, Integrated Traditional Chinese and Western Medicine Hospital of Changsha, Changsha Hospital of Hunan Normal University, Changsha, China

**Keywords:** severe anti-NMDAR encephalitis, intravenous immunoglobulin (IVIg), protein A immunoadsorption, modified rankin scale, clinical assessment scale for autoimmune encephalitis score

## Abstract

**Objective:**

To compare the differential effects of intravenous immunoglobulin (IVIg) and protein A immunoadsorption (PAIA) on neurological functional improvement in patients with severe anti-NMDAR encephalitis.

**Methods:**

We retrospectively evaluated patients with severe anti-NMDAR encephalitis (modified Rankin scale, mRS ≥ 3) at the Second People’s Hospital of Hunan from January 1, 2019, to December 31, 2024. Clinical efficacy was compared between the IVIg and PAIA groups. Clinical improvement (ΔmRS ≥ 1) and favorable functional outcomes (mRS 0-2) at 30 days and 90 days were evaluated as primary outcomes. Secondary outcomes included changes in mRS and the Clinical Assessment Scale for Autoimmune Encephalitis (ΔCASE) at 30 and 90 days, length of ICU stay, and antibody titers in cerebrospinal fluid (CSF) and serum.

**Results:**

This study enrolled 53 patients with severe anti-NMDAR encephalitis, of whom 30 patients received IVIg and 23 patients received PAIA. The PAIA group showed a significantly higher rate of clinical improvement at 30 days after treatment compared to the IVIg group (82.61% *vs*. 50%, *p* = 0.014). However, no significant difference was found at 90 days after treatment (95.65% *vs.* 96.67%, *p* > 0.05). Furthermore, favorable functional outcomes at 30 days (17.39% *vs.* 6.67%) and 90 days (91.30% *vs.* 80.00%) showed no significant differences between the two groups (*p* > 0.05). Significant differences were observed in ΔmRS_1_ (*p* = 0.005), ΔmRS_2_ (*p* = 0.03), and ΔCASE_1_ (*p* = 0.027), but not in ΔCASE_2_ (*p* > 0.05). PAIA was associated with a greater reduction in antibody titers in both CSF and serum and a shorter ICU stay.

**Conclusion:**

Our study demonstrates that both IVIg and PAIA are effective treatments for patients with severe anti-NMDAR encephalitis. However, PAIA demonstrates several distinct advantages, including earlier clinical improvement, faster antibody clearance, and a potential reduction in ICU stay.

## 1 Introduction

Autoimmune encephalitis comprises a group of non-infectious, immune-mediated inflammatory disorders of the brain and has been identified as the third most frequent cause of encephalitis, following infections and acute disseminated encephalomyelitis (1). As the most common type of AE, anti-N-methyl-D-aspartate receptor (NMDAR) encephalitis is primarily recognized as a B cell-dependent autoimmune disease, mainly caused by antibodies targeting neuronal cell-surface receptors and mediating neuronal dysfunction through direct interaction with the target antigen (2, 3). Typical clinical manifestations of patients with anti-NMDAR encephalitis include abnormal psychiatric behavior or cognitive dysfunction, speech dysfunction, seizure, involuntary movement, autonomic dysfunction or central hypoventilation, and decreased level of consciousness (4). A retrospective analysis revealed that anti-NMDAR encephalitis accounted for 1% of all intensive care unit (ICU) admissions among young adults and often requires prolonged hospitalization, which leads to a considerable social burden (5). Anti-NMDAR antibodies, which target the GluN1 subunit of the NMDAR, are predominantly of the IgG1 subclass within the IgG category (6). Accurate diagnosis of anti-NMDAR encephalitis relies crucially on the detection of specific antibodies in cerebrospinal fluid (CSF), as these antibodies are key markers of the disease (7).

Prompt treatment is essential for achieving better outcomes and reducing the frequency of relapses (8, 9). The current treatment recommendations mainly aim to eliminate circulating antibodies and address underlying immunologic triggers, such as teratomas or other tumors (3). According to previous studies, immunotherapy for anti-NMDAR encephalitis mainly includes intravenous methylprednisolone (IVMP), intravenous immunoglobulin (IVIg), plasma exchange (PE), or immunoadsorption (IA) as first-line therapies, and rituximab, tocilizumab, ofatumumab, or cyclophosphamide as second-line drugs in refractory cases (10–12). Glucocorticoids are frequently employed to inhibit the inflammatory process of disease. However, they have a lower degree of specificity and limited efficacy in cases of AE. Therefore, glucocorticoids are often administered in combination with IVIg or PE as first-line agents (13). Besides, although IVIg is easy to administer and often used as the initial treatment for AE, previous studies on its efficacy and safety have indicated that only 44% of AE patients experienced improvement within 4 weeks of IVIg therapy in conjunction with glucocorticoids (13, 14). PE is effective in reducing the concentration of autoantibodies and other pathogenic substances in the circulation (8). However, its availability is limited in many hospitals due to the insufficient supply of fresh frozen plasma. Thus, despite being a treatable condition with numerous treatment options available, there are not enough clinical trials for the treatment of AE, and current recommendations are primarily based on retrospective studies and expert opinions. Protein A immunoadsorption (PAIA), a refined form of apheresis technique using adsorption columns containing Staphylococcus aureus protein A to remove immunoglobulin G (IgG) and immune complexes, has demonstrated its capacity to remove pathogenic antibodies more quickly and accelerate recovery compared to steroids and IVIg (15). Moreover, PAIA offers the advantages of good patient tolerance and a lower risk of allergic complications compared to PE (16). Recent studies have consistently shown the efficacy of PAIA in treating a range of neuroimmune disorders, such as Guillain-Barré syndrome, myasthenia gravis, neuromyelitis optica spectrum disorder, and anti-NMDAR encephalitis (6) (15, 17). However, the sample sizes of those studies were small and there was a lack of solid clinical trials comparing the efficacy and safety of IVMP combined with IVIg versus PAIA in severe anti-NMDAR encephalitis patients. Thus, we conducted this study.

## 2 Materials and methods

### 2.1 Study design

**Figure 1:**
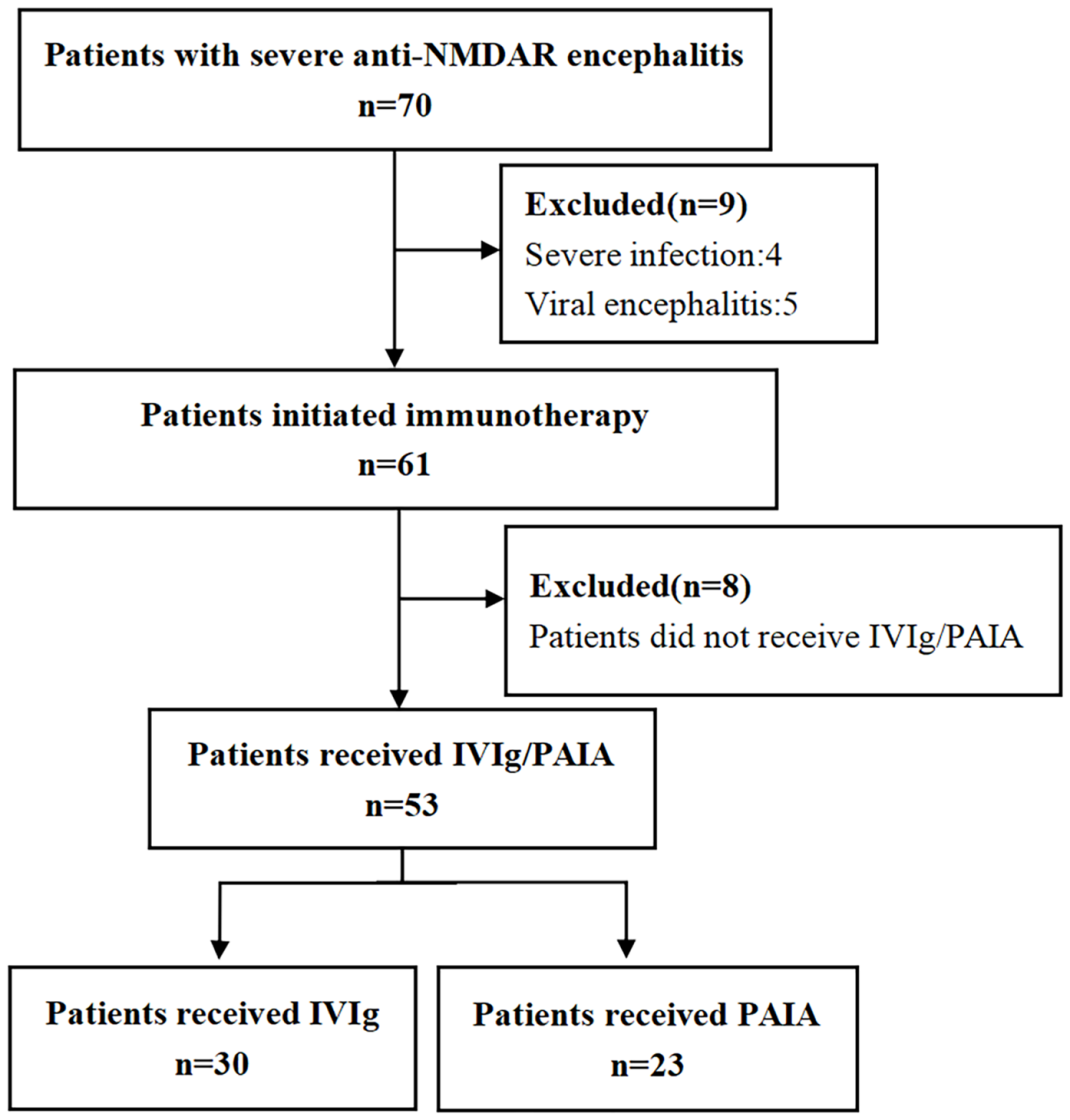
The enrollment workflow and the group assignment of patients with anti-NMDAR encephalitis in the present study. NMDAR, N-methyl-D-aspartate receptor; IVIg, intravenous immunoglobulin; PAIA, protein A immunoadsorption.

This study retrospectively analyzed patients with severe anti-NMDAR encephalitis who underwent IVIg or PAIA at the Second People’s Hospital of Hunan from January 1, 2019, to December 31, 2024. The diagnostic criteria for anti-NMDAR encephalitis were as follows: (1) positive anti-NMDAR antibodies in cerebrospinal fluid; (2) at least one or more of the major groups of anti-NMDAR encephalitis symptoms, including abnormal psychiatric behavior or cognitive dysfunction, speech dysfunction, seizure, involuntary movement, autonomic dysfunction or central hypoventilation, and decreased level of consciousness (18). The inclusion criteria were as follows: (1) patients who received a diagnosis of anti-NMDAR encephalitis according to the diagnostic criteria above; (2) admitted to the neurological ICU with severe disability (The Modified Rankin Scale score ranging from 3 to 5), including respiratory failure necessitating mechanical ventilation, altered consciousness, status dystonicus or status epilepticus; (3) received IVMP within 1 month after encephalitis onset; (4) received IVIg or PAIA in the acute phase (≤1 month after encephalitis onset). Exclusion criteria included patients with other neurological diseases, severe systemic infection, or concurrent malignancy, with the exception of ovarian teratomas. The screening process is visually depicted in **Figure 1**.

### 2.2 Ethical considerations

The study was reviewed and approved by the Ethics Committee of the Second People’s Hospital of Hunan (IRB Approval No. K2025043; Date: 2025-02-10) and was conducted in accordance with the Declaration of Helsinki. Written informed consent was obtained from all participants or their legal guardians. The consent process included a detailed explanation of the study’s objectives, procedures, potential risks and benefits, confidentiality measures, and the voluntary nature of participation. Participants were given ample opportunity to ask questions and were informed of their right to withdraw from the study at any time without penalty. For minors or incapacitated individuals, consent was obtained from parents or guardians, and assent was obtained from children aged ≥12 years. Signed consent forms were securely stored in accordance with institutional data protection policies.

### 2.3 Treatment protocol

Tumor resection was performed in complicated cases involving the tumor. All patients without contraindication received IVMP therapy combined with IVIg or PAIA. Methylprednisolone Sodium Succinate for Injection (Nang Kuang Pharmaceutical Co., Ltd, China) was administered at a dosage of 1000 mg per day for 3 days, followed by 500 mg per day for 3 days, and then 80 mg per day for 2 weeks. Human immunoglobulin (Nan Yue Biopharming Co., Ltd, China) was administered at a dosage of 0.4 g/kg per day for each course, which lasted for 5 days. PAIA was performed using a protein A IA column (KONPIA^®^; Guangzhou Koncen Bioscience Co., Ltd. Product type: KCIA08) every 2 to 3 days. Each adsorption cycle involved the regeneration of 3600 to 6000 ml of plasma, with anticoagulation achieved through heparinization. Based on the physician’s evaluation, human immunoglobulin (2.5–5 g, Nan Yue Biopharming Corporation LTD, China) was administered as an alternative therapy whenever the post-adsorption serum IgG concentration dropped below 5 g/L. One PAIA treatment course was defined as at least three therapeutic IA sessions, or determined according to the doctor’s judgment.

### 2.4 Data collection

The clinical data of all patients were retrospectively compared and analyzed, including demographic data, clinical characteristics, auxiliary examinations, anti-NMDAR antibodies, the time from onset to treatment, adverse reactions, the length of ICU stay, and other related data. The mRS score and Clinical Assessment Scale for Autoimmune Encephalitis (CASE) score were assessed before the initiation of immunotherapy and at 30 days and 90 days after the initiation of immunotherapy. The clinical efficacy in improving neurological function was determined by the rate of neurological improvement, with ΔmRS≥1 indicating functional improvement and mRS 0–2 indicating favorable functional outcomes. We also compared the mRS and CASE scores between the IVIG group and the PAIA group at 30 days and 90 days after treatment, with ΔmRS_1_=(the mRS score at 30 days after treatment)-(the mRS score at baseline), ΔmRS_2_=(the mRS score at 90 days after treatment)-(the mRS score at baseline), ΔCASE_1_=(the CASE score at 30 days after treatment)-(the CASE score at baseline), and ΔCASE_2_=(the CASE score at 90 days after treatment)-(the CASE score at baseline). Additionally, the anti-NMDAR antibodies in the serum and CSF were rechecked after the treatment. The numerical values of the titers, such as 10 and 32 in the examples of 1:10 and 1:32, were employed for analysis. A decrease in these numerical values indicates a reduction in the patient’s antibody titer.

### 2.5 Statistical analysis

Statistical analyses were performed using the statistical software SPSS 29.0 (IBM Corporation, Armonk, NY). The Shapiro-Wilk test was used to check the normality of continuous data. Normally distributed data were expressed as the mean ± SD, whereas non-normally distributed data as the median (Q1, Q3). For within-group comparisons, paired-sample t-tests or Wilcoxon signed-rank tests were employed, depending on the distribution of the data. Student’s t-test, Mann-Whitney U test, or chi-squared test were used for inter-group comparisons, as appropriate. Dichotomous data were analyzed using either the chi-squared test, Yates’ corrected chi-squared test, or fisher’s exact test based on the expected frequency. For within-group analysis of clinical efficacy, comparing pre- and post-treatment outcomes, the following statistical approach was used based on the normality of the difference scores: if the differences were normally distributed, paired t-test was selected; otherwise, the Wilcoxon signed-rank test was used. All analyses were two-tailed, and a *p* < 0.05 was considered statistically significant. Bonferroni correction was used when two pairwise comparisons were made and the significance level was adjusted to α=0.025.

## 3 Results

### 3.1 Patient characteristics

A total of 70 patients diagnosed with severe anti-NMDAR encephalitis between January 1, 2019, to December 31, 2024 were initially screened, of whom four patients with severe infection, five patients with viral encephalitis, and eight patients who did not receive IVIg/PAIA were excluded from the study based on the exclusion criteria above (**Figure 1**). Finally, 53 patients who initiated immunotherapy within 4 weeks following the onset of encephalitis were enrolled in this study, including 18 males (33.96%) and 35 females (66.04%). All of those patients were administered IVMP combined with either IVIg or PAIA during the acute phase of encephalitis. 30 patients received IVIg and 23 patients received PAIA in the acute phase of encephalitis. The two groups were well-matched in terms of sex distribution, age, and disease duration (*p*>0.05). There were no significant differences between the two groups in baseline mRS scores, CASE scores, clinical manifestations, CSF profiles, and anti-NMDAR antibody titers before treatment (*p*>0.05). Abnormal cerebral magnetic resonance imaging (MRI) findings were observed in 7/30 (23.3%) patients in the IVIg group and in 5/23 (21.7%) patients in the PAIA group (*p*>0.05). Abnormal electroencephalogram(EEG) findings, including slow-wave activity and epileptic discharge, were observed in 25/30 (83.3%) patients in the IVIg group and in 20/23 (87.0%) patients in the PAIA group (*p*>0.05). No significant difference was found in teratoma incidence (2/30 *vs* 3/23, *p*>0.05) between the two groups (**Table 1**).

**Table 1:** Characteristics of patients with anti-NMDAR encephalitis at baseline.

Baseline characteristics	Total *N=53*	IVIg *N=30*	PAIA *N=23*	*P* value	t/Z/χ²
Gender, *n* (%):				0.912	0.012
Male	18 (33.96%)	10 (33.33%)	8 (34.78%)		
Female	35 (66.04%)	20 (66.67%)	15 (65.22%)		
Age, year, median (Q1,Q3)	26.00[18.00;35.00]	31.50[18.25;39.50]	21.00[16.00;31.50]	0.096	-1.664
Disease Duration, median (Q1,Q3)	7.00 [5.00;14.0]	7.00 [5.00;13.0]	10.0 [5.00;13.0]	0.570	-0.568
mRS score at baseline, median (Q1,Q3)	5.00 [4.00;5.00]	4.00 [3.00;5.00]	5.00 [4.00;5.00]	0.216	-1.236
CASE score at baseline, mean (± SD)	16.32 (4.96)	16.17 (5.36)	16.52 (4.50)	0.799	-0.256
CSF antibody titer before IVIg/PAIA, (1:n), *n* (%)	32.00 [10.00;100.00]	32.00 [10.00;100.00]	32.00 [10.00;32.00]	0.675	-0.419
Serum antibody titer before IVIg/PAIA, (1:n), *n* (%)	32.00 [10.00;100.00]	10.00 [10.00;100.00]	32.00 [10.00;100.00]	0.203	-1.273
Clinical manifestations, *n*(%)					
Abnormal psychiatric behavior, *n* (%)	49 (92.45%)	27 (90.00%)	22 (95.65%)	0.805	0.061
Disorders of consciousness, *n* (%)	38 (71.70%)	20 (66.67%)	18 (78.26%)	0.353	0.862
Seizure, *n* (%)	38 (71.70%)	23 (76.67%)	15 (65.22%)	0.359	0.841
Cognitive dysfunction, *n* (%)	34 (64.15%)	21 (70.00%)	13 (56.52%)	0.311	1.028
speech dysfunction, *n* (%)	41(77.4%)	22(73.3%)	19(82.6%)	0.424	0.639
Involuntary movement, *n* (%)	27 (50.94%)	14 (46.67%)	13 (56.52%)	0.477	0.506
Autonomic dysfunction, *n* (%)	30 (56.60%)	18 (60.00%)	12 (52.17%)	0.569	0.325
CSF profiles					
Intracranial pressure, mmH_2_O, median (Q1,Q3)	160 [140;190]	170 [146;184]	150 [118;225]	0.272	-1.097
WBC, ×10^6^/L, median (Q1,Q3)	14.0 [4.00;31.0]	13.5 [3.25;29.8]	16.0 [4.00;27.0]	0.993	-0.009
Pro, mg/L, median (Q1,Q3)	302.00 [245.60;366.80]	319.60 [275.45;393.22]	273.50 [225.70;343.85]	0.178	-1.347
Glu, mmol/L, median (Q1,Q3)	3.86 [3.43;4.10]	3.66 [3.41;4.09]	3.92 [3.54;4.10]	0.282	-1.077
Lac, mmol/L, median (Q1,Q3)	1.73 [1.51;2.08]	1.78 [1.66;2.18]	1.68 [1.48;1.98]	0.281	-1.077
Brain MRI abnormality, *n* (%)	12 (22.6%)	7 (23.3%)	5 (21.7%)	0.891	0.019
EEG abnormality, *n* (%)	45 (84.9%)	25 (83.3%)	20 (87.0%)	1.000	0
Teratoma, *n* (%)	5 (9.43%)	2 (6.67%)	3 (13.04%)	0.754	0.098

NMDAR, N-methyl-D-aspartate receptor; mRS, modified Rankin scale; CASE, Clinical Assessment Scale for Autoimmune Encephalitis; CSF, cerebrospinal fluid; WBC, white blood cell; Pro, protein; Glu, glucose; Lac, lactic acid; MRI, magnetic resonance imaging; EEG, electroencephalogram; IVIg, intravenous immunoglobulin; PAIA, protein A immunoadsorption.

The time from disease onset to initiation of immunotherapy and the time from disease onset to initiation of IVMP showed no significant difference (*p*>0.05). The median duration from the onset to the initiation of IVIg was 15.57 (± 5.35) days, and from the onset to the initiation of PAIA was 16.7 (± 6.17) days (*p*>0.05). The median number of IVIg treatment courses was 1[1;1]. The average number of sessions per patient in the PAIA group was 5[5;6]. The total number of treatment sessions for the PAIA group was 126. The average volume of plasma regenerated per session was 3600 to 6000 ml for the PAIA group. Rituximab (2/30 in the IVIg group *vs* 1/23 in the PAIA group), ofatumumab (2/30 in the IVIg group *vs* 0/23 in the PAIA group) were used as second-line therapies in both groups. Tumors in all 5 patients were completely removed by surgery. All patients were followed up for a period of 3 months and administered oral steroids (**Table 2**).

**Table 2:** Details of immunotherapy and comparison of clinical efficacy between IVIg and PAIA.

Baseline characteristics	Total *N=53*	IVIg *N=30*	PAIA *N=23*	*P* value	t/Z/χ²
Days between onset and immunotherapy, mean (± SD)	15.28 (5.31)	15.17 (4.80)	15.43 (6.03)	0.858	-0.180
Days between onset and IVMP, median (Q1,Q3)	16.0 [11.0;20.0]	16.5 [12.5;20.0]	16.0 [10.0;21.5]	0.957	-0.054
Days between onset and IVIg/PAIA, mean (± SD)	16.06 (5.69)	15.57 (5.35)	16.70 (6.17)	0.48	-0.712
Course of IVIg per patients, median(Q1,Q3)		1[1;1]	–	–	
Number of treatment sessions per patients, median (Q1,Q3)	–	–	5[5;6]	–	
Second-line therapy; *n*(%)	5(9.43%)	4(13.33%)	1(4.34%)		
Rituximab; *n*(%)	3(5.66%)	2(0.66%)	1(4.34%)	–	
Ofatumumab; *n*(%)	2(3.77%)	2(0.66%)	0	–	
Days between immunotherapy and retesting antibodies, median (Q1,Q3)	16[14;19]	16[14;20.25]	16[14;18]	0.549	-0.600
The rate of decline in CSF antibody titers; *n*(%)	33(62.3%)	14(46.7%)	19(82.6%)	**0.007**	7.158
The rate of decline in serum antibody titers; *n*(%)	33(62.3%)	15(50.0%)	18(78.3%)	**0.035**	4.425
Clinical improvement at 30 days, *n*(%)	34 (64.15%)	15 (50.00%)	19 (82.61%)	**0.014**	6.019
Clinical improvement at 90 days, *n*(%)	51 (96.23%)	29 (96.67%)	22 (95.65%)	1	1.000
Favorable functional outcomes at 30 days, *n*(%)	6 (11.32%)	2 (6.67%)	4 (17.39%)	0.433	0.615
Favorable functional outcomes at 90 days, *n*(%)	45 (84.91%)	24 (80.00%)	21 (91.30%)	0.452	0.566
ICU stay, days, median (Q1,Q3)	24.00[19.00;30.00]	27.00[20.00;30.75]	22.00[16.50;24.50]	**0.03**	-2.176
Hospital stay, days, median (Q1,Q3)	36.00 [33.00;41.00]	37.50[33.25;41.00]	35.00[32.00;40.00]	0.301	-1.033

IVMP, intravenous methylprednisolone; IVIg, intravenous immunoglobulin; PAIA, protein A immunoadsorption; CSF, cerebrospinal fluid; mRS, modified Rankin scale; CASE, Clinical Assessment Scale for Autoimmune Encephalitis; ICU, Intensive Care Unit. The bold values provided indicate statistical significance (p < 0.05).

### 3.2 Clinical efficacy of IVIg versus TPE

**Figure 2:**
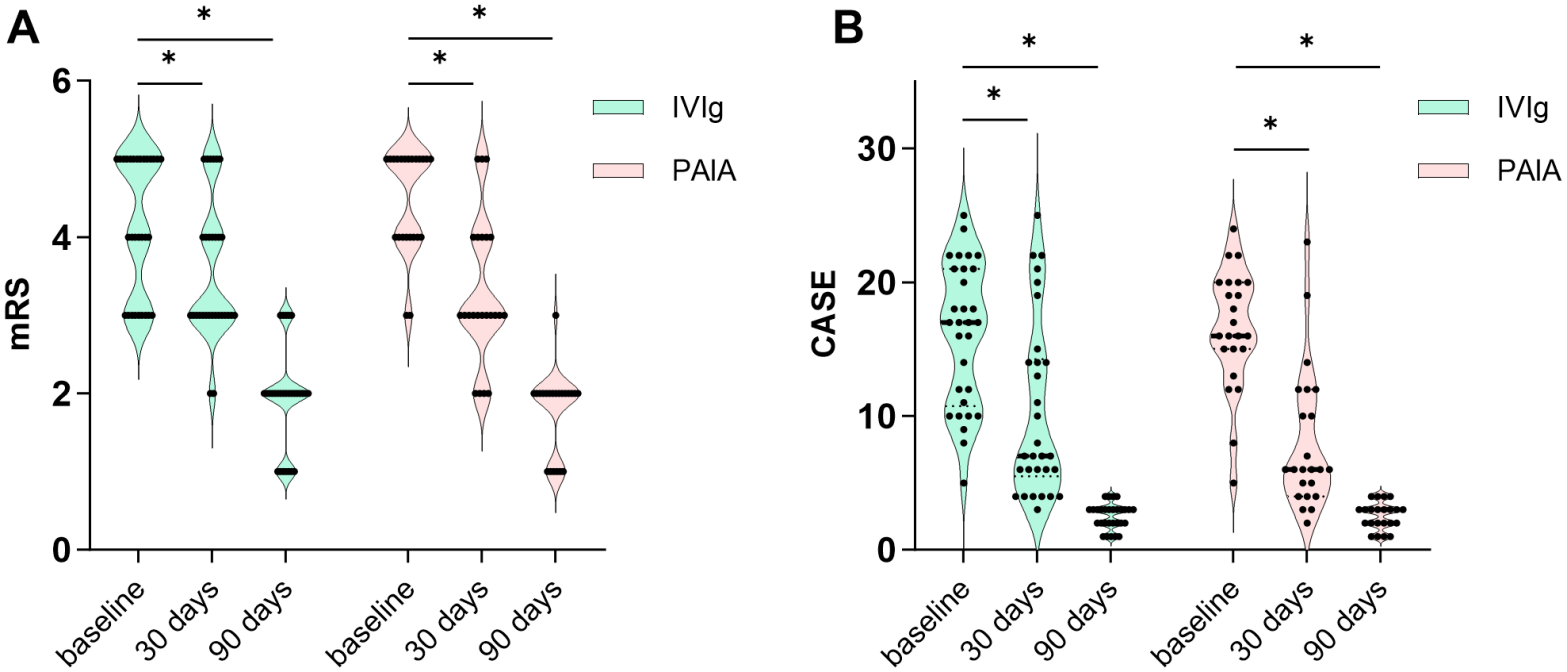
Clinical efficacy of IVIg and PAIA. **(A)** Significant decreases in mRS scores were observed at 30 days and 90 days after IVIg treatment (30 days, Z = -3.494, *p* < 0.001; 90 days, Z = -4.562, *p* < 0.001) and PAIA treatment (30 days, Z = -3.852, *p* < 0.001; 90 days, Z = -4.178, *p* < 0.001). **(B)** Significant decreases in CASE scores were also observed at 30 days and 90 days after IVIg treatment (30 days, t = 7.762, *p* < 0.001; 90 days, t = 11.949, *p* < 0.001) and PAIA treatment (30 days, t = 8.782, *p* < 0.001; 90 days, Z = -4.079, *p* < 0.001). mRS, modified Rankin scale; CASE, Clinical Assessment Scale for Autoimmune Encephalitis; IVIg, intravenous immunoglobulin; PAIA, protein A immunoadsorption. *P<0.025.

**Figure 3:**
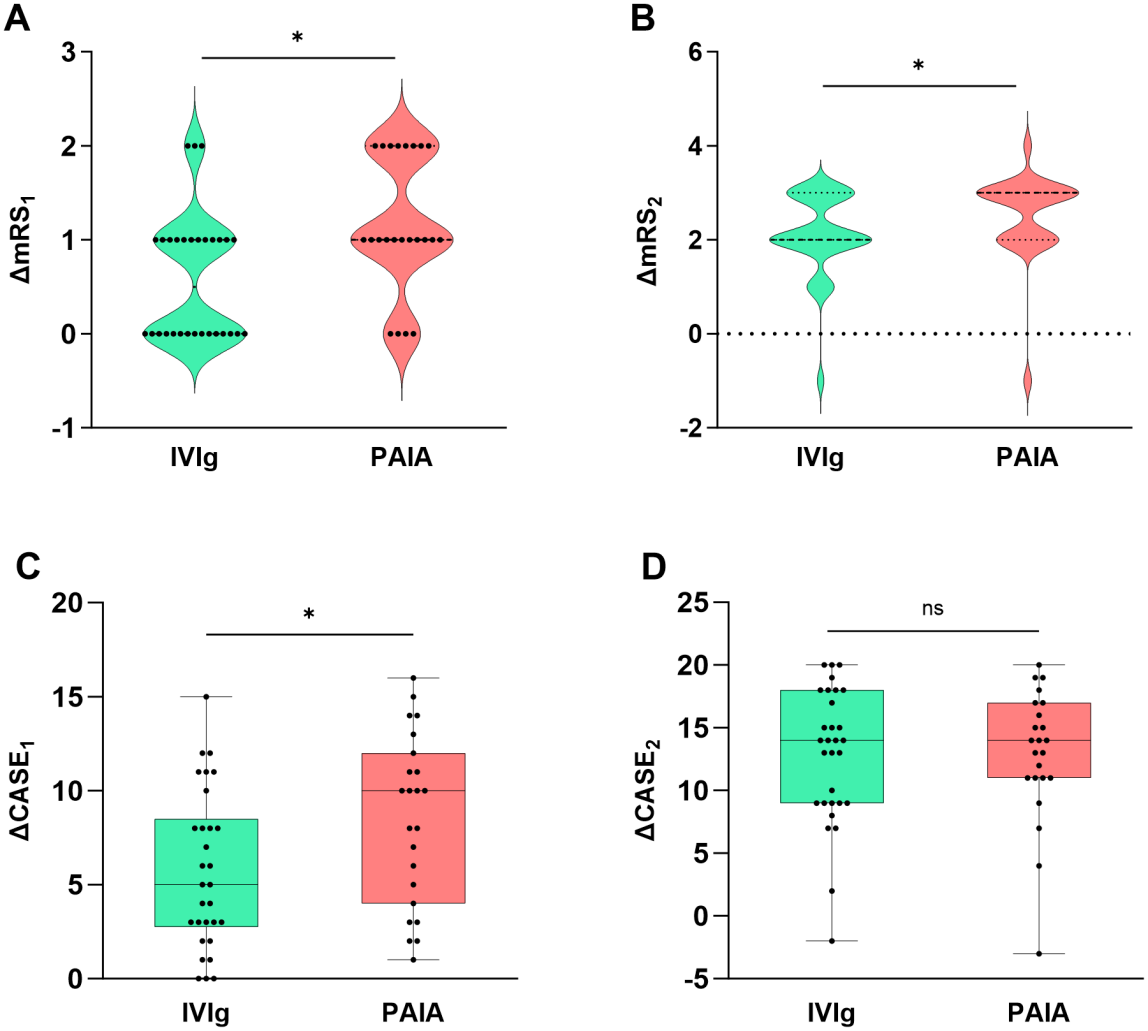
Comparison of clinical efficacy between PAIA and IVIg. There were significant differences in ΔmRS_1_ (Z = -2.781, *p* = 0.005), ΔmRS_2_ (Z = -2.168, *p* = 0.03) and ΔCASE_1_ (t = -2.283, *p* = 0.027) after treatment **(A–C)**, but ΔCASE_2_ (Z = -2.168, *p* = 0.943) showed no significant difference between the IVIg group and the PAIA group **(D)**. mRS, modified Rankin scale; CASE, Clinical Assessment Scale for Autoimmune Encephalitis; IVIg, intravenous immunoglobulin; PAIA, protein A immunoadsorption. *P<0.05, ns, no significant.

There were no significant differences in mRS scores and CASE scores between the two groups at baseline (*p* > 0.05, **Table 1**). Significant decreases in mRS score and CASE score were observed at 30 days and 90 days both in the IVIg group and the PAIA group after treatment (*p* < 0.05, **Figure 2**). Besides, PAIA had a higher rate of clinical improvement (ΔmRS≥1) at 30 days after treatment compared to IVIg (82.61% *vs.* 50%, *p*=0.014, **Table 2**). However, no significant difference was found at 90 days after treatment(96.67% *vs.* 95.65%, *p*>0.05,**Table 2**). Furthermore, we compared the favorable functional outcomes(mRS 0-2) at 30 days(6.67% *vs.* 17.39%) and 90 days(80.00% *vs.* 91.30%) between the IVIg group and the PAIA group and also found no significant differences(*p*>0.05; **Table 2**). There were significant differences in ΔmRS_1_(*p*= 0.005), ΔmRS_2_(*p*= 0.03) and ΔCASE_1_ (*p*=0.027) after treatment, but ΔCASE_2_ (*p*> 0.05) showed no significant difference between the IVIg group and the PAIA group (**Figure 3**). We observed a 46.7% reduction in CSF antibody titers in the IVIg group and an 82.6% reduction in the PAIA group (*p*=0.007). Additionally, we observed a 50% reduction in serum antibody titers in the IVIg group and a 78.3% reduction in the PAIA group (*p*=0.035), after 16[14;19] days of treatment.

The length of ICU admission showed significant differences between the IVIg group and the PAIA group(HL median difference: 5days, 95% CI [0, 8], p=0.03). The total hospital stay showed no significant differences(HL median difference: 2days, 95% CI [-2, 6],p=0.301).

### 3.3 Safety

In the present study, the reported adverse events associated with IVIg were allergic reactions, which occurred in 3 out of 30 patients (10%). Regarding PAIA, transient hypotension occurred during 20 out of 126 sessions (15.8%) and required a fluid bolus or vasopressor treatment. Extracorporeal system coagulation occurred in 1 session, and rupture of the plasma separator membrane occurred in 1 session; both required discontinuation of PAIA. Other potential adverse effects of PAIA, such as arrhythmia, infection, hemorrhage, and hemolysis, were not observed. The incidence of adverse events was 17.4% (20/126 sessions). Notably, no serious adverse events or treatment-related deaths were detected.

## 4 Discussion

This retrospective study aimed to compare the efficacy and safety of IVIg and PAIA in improving neurological function in patients with severe anti-NMDAR encephalitis, with the goal of identifying more effective therapeutic strategies. mRS and CASE are two important tools for assessing the severity of autoimmune encephalitis, but each has its limitations. mRS is commonly used to assess neurological function recovery in patients after stroke and is limited in assessing AE severity due to its focus on motor function, narrow scoring range, poor correlation with cognitive outcomes, and inadequacy in longitudinal monitoring (17, 19, 20). CASE is another sensitive tool for evaluating clinical improvement in patients with AE (17). It performs better for non-motor symptoms and is more sensitive to changes in severity than the mRS, but its application in severe AE patients is limited (21, 22). Thus, we combined these two tools to evaluate the efficacy of treatment in AE patients.

Our study assessed the response to IVIg and PAIA by examining changes in mRS scores and CASE scores at baseline, 30 days, and 90 days after treatment. We observed a significant decrease in mRS scores and CASE scores at 30 days and 90 days post-treatment in both groups, indicating that both IVIg and PAIA are effective treatments for severe anti-NMDAR encephalitis, as previously demonstrated in prior studies (10, 16). Then, we compared the rates of clinical improvement and favorable functional outcomes at 30 days and 90 days between the IVIg and PAIA groups. The clinical improvement rate at 30 days in the IVIg group was 50%, which was comparable to those reported in previous studies (13). In contrast, we observed that 82.6% of patients in the PAIA group exhibited clinical improvement at 30 days, which was slightly higher than the rates reported in previous studies (15, 16). Specifically, those studies revealed that 77.78% of patients receiving PAIA demonstrated significant clinical improvement at 30 days, and 94.4-100% at 3 months (15, 16). The clinical improvement rate was significantly higher in the PAIA group at the 30-day follow-up compared to the IVIg group. However, the difference in clinical improvement at the 90-day follow-up was not significant. These statistics may indicate that PAIA therapy is more effective than IVIg in achieving earlier clinical improvement.

No significant difference was observed in the rate of favorable functional outcomes between the two groups at either 30 days or 90 days. These findings may be related to the following reasons: 1) The sample size of the study was relatively small; 2) The study subjects were critically ill patients, and it was difficult for them to achieve a favorable functional outcome within 30 days; 3) Due to early standardized and effective treatment, most patients in both groups achieved a favorable functional outcome by the 90-day follow-up.

The reductions in mRS scores and CASE scores were indicated by ΔmRS_1_, ΔmRS_2_, ΔCASE_1_, and ΔCASE_2_. We also observed greater reductions in mRS scores and CASE scores in the PAIA group at 30 days. This may indicate that PAIA had a greater advantage in rapidly improving patients’ clinical symptoms in the early stage. We attempted to explain this conclusion with the following points. The primary mechanism of action in IVIg is the neutralization of autoantibodies (13). However, anti-NMDAR antibodies primarily belong to the IgG1 subclass of IgG, while IVIg may also react with other types of immunoglobulins simultaneously, thereby reducing the therapeutic effect. PAIA could directly remove IgGs, especially IgG1, 2, and 4, due to the affinity of recombinant staphylococcal protein A for the Fc fragment of IgG (23). This may lead to the rapid clearance of antibodies, and more favorable short-term benefits. The decrease in serum antibody titers also substantiates these viewpoints. Consistently, we observed a significant reduction in serum and CSF antibody titers in the PAIA group compared with the IVIg group. This observation is supported by a previous report on the use of IA to treat AE, which found that CSF antibody titers reduced in 64% of patients, measured at a median of 5 days following the last IA session (24). These results indicate that the removal of systemic antibodies via PAIA effectively reduces CSF antibody levels, likely due to redistribution across the damaged blood-brain barrier.

However, there was a significant difference in mRS score reductions between the two groups at 90 days after treatment, but not in CASE scores. This suggests that CASE scores may not be sensitive enough to measure meaningful clinical responses in anti-NMDAR encephalitis patients at 90 days. Future studies with larger sample sizes are needed to confirm this observation.

The Chinese Expert Consensus on the Diagnosis and Management of Autoimmune Encephalitis (2022 edition) recommends that second-line immunotherapy should be promptly initiated if there is no significant improvement in the condition after 2 weeks of first-line immunotherapy. Notably, in our study, second-line immunotherapy was administered more than 30 days after disease onset in all five patients. Therefore, it did not influence the 30-day outcomes. Regarding the 90-day outcomes, we excluded patients who had received second-line immunotherapy from both groups and re-performed statistical analyses for clinical improvement at 90 days, favorable functional outcomes at 90 days, ΔmRS_2_ and ΔCASE_2_. The results were consistent with our previous findings (**Supplementary Materials 1 and 2**). Therefore, the use of second-line agents may have enhanced overall efficacy but did not influence the efficacy evaluation of IVIg and PAIA in our study.

A retrospective study revealed that 77% of patients with AE require ICU support, mostly due to respiratory failure necessitating mechanical ventilation, disorders of consciousness, or status epilepticus (14, 25). Some previous studies have found that ICU admission is an independent predictor of poor functional outcomes for patients with anti-NMDAR encephalitis (14, 26).The duration of ICU stay for patients with AE is influenced by a combination of factors, including disease severity, timeliness of treatment, complications, patient baseline characteristics, and availability of medical resources. Shortening the ICU duration is also a therapeutic goal, both for managing the disease itself and for reducing the economic burden. We observed that patients undergoing PAIA had a relatively shorter ICU stay, which may alleviate patient suffering, shorten the disease course, and reduce complications.

Additionally, we compared the safety of IVIg and PAIA. The most common side effects of IVIg were allergic reactions. In contrast, PAIA patients experienced a higher incidence of adverse events, including hypotension, extracorporeal system coagulation, and rupture of the plasma separator membrane during the procedure. These could be related to the longer duration of each session and the placement of central venous catheters. To shorten the duration of each course and to use anticoagulant drugs when necessary may help address these issues.

While providing valuable clinical insights, our study has several limitations. First, as a single-center retrospective analysis, the observational design precludes causal inferences. Second, the small sample size and age disparity between groups (younger PAIA participants) suggest potential selection bias, possibly reducing statistical power (as evidenced by non-significant 90-day recovery rates) and generalizability. Additionally, population heterogeneity in disease severity and concomitant therapies complicates interpretation. Other limitations include: (1) possible recall/documentation biases, (2) restricted external validity from single-center recruitment, and (3) unmeasured confounders (e.g., rehabilitation protocols). These constraints collectively underscore the need for cautious interpretation. Future multicenter prospective studies with larger cohorts and standardized protocols are needed to validate the comparative efficacy of IVIg and PAIA therapies.

## 5 Conclusion

Our preliminary findings suggest that both IVIg and PAIA may be viable treatment options for patients with anti-NMDAR encephalitis, with PAIA showing potential advantages in terms of earlier clinical improvement, faster antibody clearance, and possible reduction in ICU stay duration. However, these observations should be interpreted with caution given the study’s retrospective design, small sample size, and other methodological limitations as discussed. Further prospective, multicenter studies with larger cohorts are needed to confirm these findings and establish definitive treatment recommendations.

## Data Availability

The original contributions presented in the study are included in the article/**Supplementary Material**, further inquiries can be directed to the corresponding author/s.
